# The Prognostic Value of New Index (LANR) Composed of Pre-operative Lymphocytes, Albumin, and Neutrophils in Patients With Resectable Colorectal Cancer

**DOI:** 10.3389/fonc.2021.610264

**Published:** 2021-05-25

**Authors:** Xinjun Liang, Shuang Yao, Ping Lu, Yifei Ma, Hongli Xu, Zhucheng Yin, Junjie Hu, Yanyan Liu, Shaozhong Wei

**Affiliations:** ^1^Department of Medical Oncology, Tongji Medical College, Hubei Cancer Hospital, Huazhong University of Science and Technology, Wuhan, China; ^2^Colorectal Cancer Clinical Research Center of Hubei Province, Wuhan, China; ^3^Colorectal Cancer Clinical Research Center of Wuhan, Wuhan, China; ^4^Department of Gastrointestinal Surgery, Tongji Medical College, Hubei Cancer Hospital, Huazhong University of Science and Technology, Wuhan, China; ^5^Division of Nephrology, Tongji Medical College, Tongji Hospital, Huazhong University of Science and Technology, Wuhan, China

**Keywords:** lymphocytes, albumin, neutrophils, colorectal cancer, prognosis

## Abstract

**Background:** Inflammatory factors and nutritional status are critical to the prognosis of colorectal cancer patients. This study aimed to investigate the prognostic value of the combination of preoperative lymphocytes, albumin, and neutrophils (LANR) in patients with resectable colorectal cancer.

**Methods:** A total of 753 patients with pathologically diagnosed primary colorectal cancer were included in the study. The value of LANR was defined as follows: LANR, lymphocyte × albumin/neutrophil. The ROC curve, subgroup analysis and Cox proportional hazard regression analysis were used to assess the prognostic value of LANR in overall survival and progression-free survival.

**Results:** The median age of the patients was 60 years (range 52–67 years). In overall survival, the area under the curve of LANR was 0.6276, and the HR (95% CI) was 0.551 (0.393–0.772). And in progression-free survival, the area under the curve of LANR was 0.5963, and the HR (95% CI) was 0.697 (0.550–0.884). The results indicate that preoperative LANR may be a reliable predictor of overall and progression-free survival in resectable colorectal cancer patients.

**Conclusions:** LANR is an important prognostic indicator for patients with resectable colorectal cancer, and it can also provide a reference for clinicians and patients to choose a treatment plan.

## Introduction

Colorectal cancer (CRC) is an important public health problem. In 2018, there were more than 1.8 million new cases of colorectal cancer and 881,000 deaths worldwide, accounting for about one-tenth of global cancer incidence and deaths. And the incidence of colorectal cancer ranks third and the mortality rate ranks second (1). From a clinical perspective, surgery has been established as the main treatment for colorectal cancer (2). Although advances in medical treatment have gradually improved the survival of patients (3). In fact, even after surgery, the prognosis of colorectal cancer is far from satisfactory (4, 5). Therefore, finding more effective biomarkers to predict the prognosis of colorectal cancer becomes particularly important.

With the continuous development of tumor prognosis research, more and more evidence indicates the role of inflammatory factors and nutritional status in cancer prognosis (6–8). Systemic inflammatory factors, such as lymphocytes (9), monocytes (10) and neutrophils (11), and blood biochemical indicators related to nutritional status, such as C-reactive protein levels (CRP) (12) and albumin levels (ALB) (13), are valuable prognostic indicators for cancers including colorectal cancer. In addition, studies have shown that the integration of these biochemical indicators such as the modified Glasgow Prognosis Score (mGPS) (14), C-reactive protein to albumin ratio (CAR) (15, 16), and neutrophil to lymphocyte ratio (NLR) (17) can effectively improve the accuracy of cancer prognosis prediction.

However, the prognostic significance of the combination of lymphocyte, albumin, and neutrophil (LANR) in colorectal cancer has not been well-investigated to date. Therefore, in this study, we retrospectively analyzed the preoperative blood biochemical indicators of 753 colorectal cancer patients, and systematically evaluated the survival prognostic value of LANR.

## Materials and Methods

### Patient Cohort

We retrospectively collected 829 patients with pathologically diagnosed primary colorectal cancer at Hubei Cancer Hospital from January 2013 to December 2016. The exclusion criteria were as follows: (1) having history of malignant tumors; (2) having incomplete clinical data; (3) having concurrent malignant tumors other than colorectal cancer; or (4) having other diseases with serious impacts on prognosis, such as ischemic heart disease and stroke. Based on the exclusion criteria, we eventually included 753 patients with colorectal cancer in our study (Supplementary Figure 1). Tumor stage was determined based on the Eighth Edition of the American Joint Committee on Cancer (AJCC) Cancer Staging Manual (18). The study was supported by the Ethics Committee and Institutional Review Board of Hubei Cancer Hospital. And all patients provided informed consent.

### Data Collection

We collected the clinicopathological information and preoperative blood biochemical indicators of patients with colorectal cancer through electronic and paper medical records from the hospital. Such as gender, age, tumor size, vascular tumor thrombus, nerve invasion, circumferential margin, radiotherapy, chemotherapy, tumor location, TNM stage, differentiation, ALB, lymphocyte, and neutrophil. Based on previous studies (19–22), we found that ALB, lymphocyte, and neutrophil were key biochemical indicators related to tumor prognosis. And we also used these three indicators to construct a new prognostic marker-LANR, which was defined as Lymphocyte × Albumin/Neutrophil. The study follow-up until August 2019. The overall survival (OS) was set as the first outcome and progression-free survival (PFS) as the secondary outcome. The PFS was defined as the time from the date of tumor resection to the date of cancer recurrence, metastasis, death, or the end of follow-up whichever came first, and the OS was calculated as the time from the date of tumor resection to the date of death or the end of follow-up.

### Statistical Analysis

The continuous variables were expressed as mean ± standard deviation ($\bar{X}$ ± SD) or median (interquartile range), and the categorical variables were presented by the number of cases (percentage). Student's *t*-test or Wilcoxon test was used to compare differences between groups of continuous variables. Chi-square or Fisher's exact tests were used to evaluate categorical variables. By using the inflection point as the cut-offs, the receiver operating characteristic (ROC) curve was used to convert continuous variables (albumin, neutrophils, lymphocytes, and LANR) into dichotomous variables. Kaplan–Meier survival curves and log-rank test were used to compare the survival difference between groups classified by dichotomized biochemical indicators. Cox proportional hazard regression model was used for univariate and multivariate regression analysis. Statistically significant variables (*P* < 0.05) in univariate analysis were included in multivariate analysis. Subgroup analysis were performed to show the prognostic association between patients with different characteristics and the new index, and the results were shown in the forest plots (23). Statistical analyses were performed using SAS 9.4 (SAS Institute Inc, Cary, North Carolina, USA) and R 3.5.1 (R Foundation for Statistical Computing, Vienna, Austria). All analyses were two-sided, and *P* < 0.05 were considered statistically significant.

### Data Availability Statement

The datasets used and/or analyzed during the current study are available from the corresponding author on reasonable request.

## Results

### Patient Characteristics

A total of 753 patients with colorectal cancer were included in this study. The demographic and clinicopathological characteristics are shown in Table 1. There were 280 females (37.18%) and 473 males (62.82%). The median age of the patients was 60 years (range 52–67 years). There were 84 patients (11.16%) in stage I, 253 (33.60%) in stage II, 274 (36.39%) in stage III, and 142 (18.86%) in stage IV. Of these patients, 452 (60.03%) had rectal tumors, and 301 (39.97%) had colon tumors. A total of 292 (38.78%) patients progressed and 145 patients (19.26%) died. The study's median progression-free survival was 63.47 months, and the median follow-up time was 37.03 months.

**Table 1:** Baseline clinicopathological characteristics of colorectal cancer patients.

		**Disease progression**		P^*^	**Death**		P^*^
		**Without**	**With**		**No**	**Yes**	
		**(*n* = 461) (%)**	**(*n* = 292) (%)**		**(*N* = 608) (%)**	**(*N* = 145) (%)**	
Age (yr)^a^		60 (51–67)	60.5 (53–67)	0.873	59 (51–67)	63 (55–68)	0.004
Sex	Male	283 (61.39)	190 (65.07)	0.316	377 (62.01)	96 (66.21)	0.39
	Female	178 (38.61)	102 (34.93)		231 (37.99)	49 (33.79)	
Tumor location	Colon	179 (38.83)	122 (41.78)	0.446	237 (38.98)	64 (44.14)	0.259
	Rectum	282 (61.17)	170 (58.22)		371 (61.02)	81 (55.86)	
TNM stage	I	73 (15.84)	11 (3.77)	<0.001	81 (13.32)	3 (2.07)	<0.001
	II	195 (42.30)	58 (19.86)		238 (39.14)	15 (10.34)	
	III	164 (35.57)	110 (37.67)		216 (35.53)	58 (40.00)	
	IV	29 (6.29)	113 (38.70)		73 (12.01)	69 (47.59)	
Tumor size (cm)	*d* < 2	12 (2.60)	2 (0.68)	0.160	12 (1.97)	2 (1.38)	0.832
	2 ≤ d <5	315 (68.33)	201 (68.84)		418 (68.75)	98 (67.59)	
	d ≥ 5	134 (29.07)	89 (30.48)		178 (29.28)	45 (31.03)	
Differentiation	Low	45 (9.76)	58 (19.86)	<0.001	72 (11.84)	31 (21.38)	0.004
	Medium	358 (77.66)	214 (73.29)		467 (76.81)	105 (72.41)	
	High	58 (12.58)	20 (6.85)		69 (11.35)	9 (6.21)	
Circumferential margin	No	459 (99.57)	280 (95.89)	<0.001	602 (99.01)	137 (94.48)	0.002
	Yes	2 (0.43)	12 (4.11)		6 (0.99)	8 (5.52)	
Vascular tumor thrombus	No	342 (74.19)	187 (64.04)	0.003	436 (71.71)	93 (64.14)	0.086
	Yes	119 (25.81)	105 (35.96)		172 (28.29)	52 (35.86)	
Nerve invasion	No	375 (81.34)	208 (71.23)	0.002	482 (79.28)	101 (69.66)	0.015
	Yes	86 (18.66)	84 (28.77)		126 (20.72)	44 (30.34)	
Chemotherapy	No	173 (37.53)	57 (19.52)	<0.001	192 (31.58)	38 (26.21)	0.229
	Yes	288 (62.47)	235 (80.48)		416 (68.42)	107 (73.79)	
Radiotherapy	No	444 (96.31)	265 (90.75)	0.002	575 (94.57)	134 (92.41)	0.326
	Yes	17 (3.69)	27 (9.25)		33 (5.43)	11 (7.59)	
ALB (G/L)^a^		42.2 (38.8–45.0)	41.6 (38.0–44.3)	0.032	42.20 (38.95–44.90)	40.44 (37.00–43.40)	<0.001
Lym (10^9^/L)^a^		1.47 (1.15–1.83)	1.33 (1.00–1.72)	0.001	1.46 (1.13–1.83)	1.29 (0.94–1.65)	<0.001
Neu (10^9^/L)^a^		3.32 (2.65–4.47)	3.89 (2.90–4.98)	0.001	3.48 (2.68–4.60)	3.93 (2.89–5.14)	0.012

*Lym, lymphocyte; ALB, albumin; Neu, neutrophils*.

* *P-values were calculated by the Student's t-test or Wilcoxon test for continuous variables, and the Chi-square test for categorical variables, respectively*.

a *Age, ALB, Lym, and Neu are continuous variables, the others (Sex, Tumor Location, TNM stage, Tumor size, Differentiation, Circumferential margin, Vascular tumor thrombus, Nerve invasion, Chemotherapy, and Radiotherapy) are categorical variables*.

### Prognostic Value of LANR in Overall Survival

**Figure 1:**
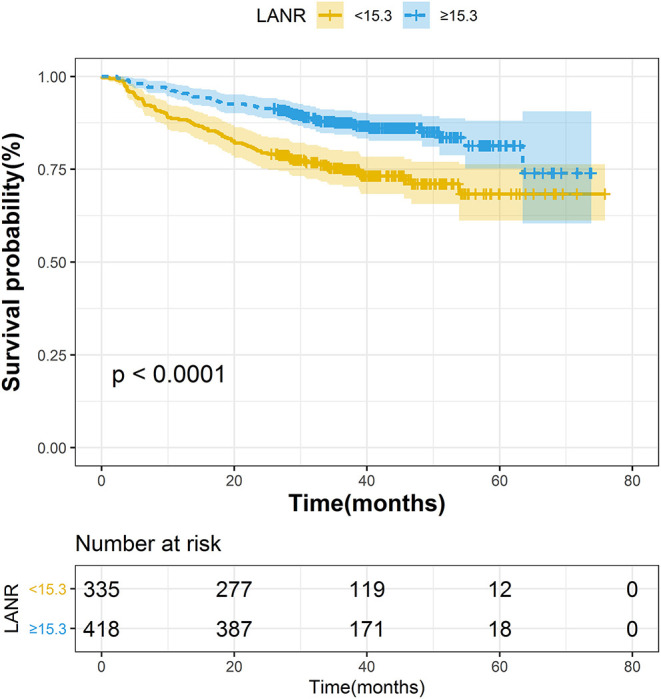
The Kaplan–Meier curves for overall survival of colorectal cancer patients based on LANR.

The areas under the ROC curves and inflection points of albumin, neutrophils, lymphocyte counts, and LANR for OS are listed in Table 2. According to the ROC curve, we found that the area under the curve of LANR was the best at 0.6276 (Supplementary Figure 2). We divided LANR into high-level (*n* = 418, 55.51%) and low-level (*n* = 335, 44.49%) groups based on cut-off values (Supplementary Table 1), and the Kaplan-Meier survival curve showed that patients with high-level LANR had longer overall survival (Figure 1). Univariate analysis revealed that TNM stage, differentiation, circumferential margin, nerve invasion, albumin, neutrophils, lymphocytes, and LANR all showed significant association with OS (all *P* < 0.05; Table 2). Multivariate analysis showed that high levels of albumin, neutrophils, lymphocytes and LANR had 0.681 (95% CI: 0.476–0.974), 1.512 (95% CI: 1.085–2.107), 0.634 (95% CI: 0.445–0.903), and 0.551 (95% CI: 0.393–0.772)-fold risk of death (Table 2). LANR presented significant associations with OS among patients in different genders, age (<65 yr), tumor locations (colon), differentiation (medium), and TNM stages (III/IV) (Supplementary Figure 3). Combining the area under the curve and the results of multivariate cox regression, we found that LANR is a valuable new prognostic indicator in overall survival.

**Table 2:** Univariate and multivariate Cox regression analysis of overall survival in patients with colorectal cancer.

	**AUC**	**Cut-Point**	**Univariate**		Multivariate^*^	
			**HR (95% CI)**	***P***	**HR (95% CI)**	***P***
ALB (G/L)	0.5968	42.8	0.562 (0.395–0.800)	0.0014	0.681 (0.476–0.974)	0.0355
Neu (10^9^/L)	0.5672	4.42	1.706 (1.226–2.373)	0.0015	1.512 (1.085–2.107)	0.0147
Lym (10^9^/L)	0.6021	1.50	0.510 (0.359–0.726)	0.0002	0.634 (0.445–0.903)	0.0116
LANR	0.6276	15.3	0.475 (0.341–0.664)	<0.0001	0.551 (0.393–0.772)	0.0005
TNM stage^#^			6.923 (4.223–11.349)	<0.0001	6.157 (3.745–10.125)	<0.0001
Age			1.488 (1.070–2.068)	0.0181	1.318 (0.944–1.841)	0.1050
Differentiation			0.564 (0.407–0.781)	0.0006	0.641 (0.462–0.889)	0.0077
Circumferential margin			3.968 (1.942–8.109)	0.0002	4.295 (2.077–8.881)	<0.0001
Nerve invasion			1.641 (1.150–2.341)	0.0063	1.625 (1.139–2.318)	0.0074

HR, Hazard Ratio; CI, Confidence Interval; AUC, Area under the ROC Curve; Lym, lymphocyte; ALB, albumin; Neu, neutrophils; LANR, Lym*Alb/Neu.

* *Multivariate cox regression models included age, TNM stage, differentiation, circumferential margin, nerve invasion, and the clinical indicators for mutual adjustment*.

# *TNM Stage adopted binary classification (I/II vs. III/IV)*.

*Reference group: TNM (I/II); Differentiation (Low); Circumferential margin (No); Nerve invasion (No)*.

### Prognostic Value of LANR in Progression-Free Survival

**Figure 2:**
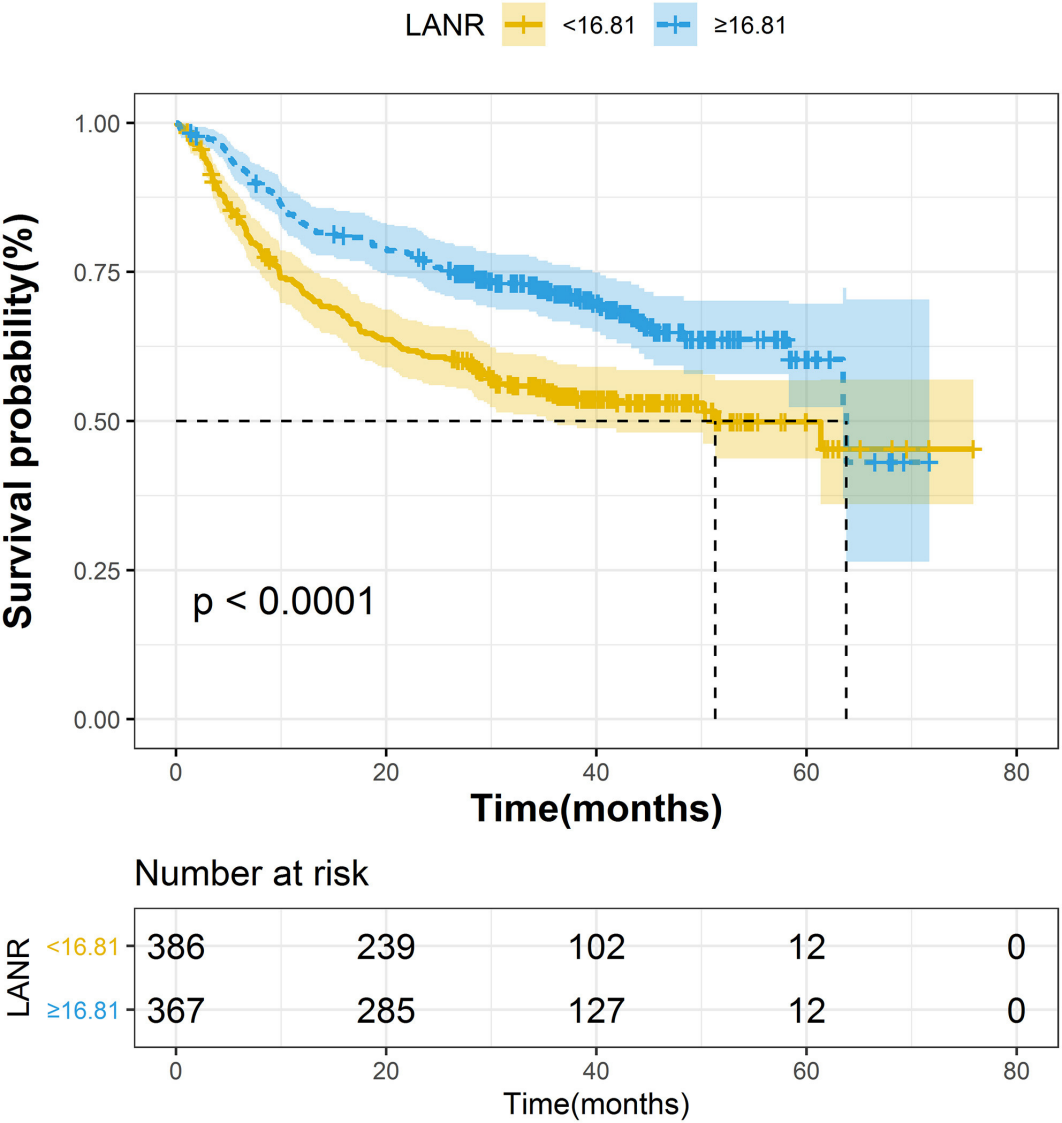
The Kaplan–Meier curves for progression-free survival of colorectal cancer patients based on LANR.

The areas under the ROC curves and inflection points of albumin, neutrophils, lymphocyte counts, and LANR for PFS are listed in Table 3. According to the ROC curve, we found that the area under the curve of LANR was the best at 0.5963 (Supplementary Figure 4). We divided LANR into high-level (*n* = 367, 48.74%) and low-level (*n* = 386, 51.26%) groups based on cut-off values (Supplementary Table 2), and the Kaplan-Meier survival curve showed that patients with high-level LANR had longer progression-free survival (Figure 2). Univariate analysis revealed that TNM stage, differentiation, circumferential margin, vascular tumor thrombus, nerve invasion, chemotherapy, radiotherapy, albumin, neutrophils, lymphocytes, and LANR all showed significant association with PFS (all *P* < 0.05; Table 3). Multivariate analysis showed that high levels of albumin, neutrophils, lymphocytes, and LANR had 0.800 (95% CI: 0.624–1.026), 1.450 (95% CI: 1.141–1.843), 0.727 (95% CI: 0.570–0.926), and 0.697 (95% CI: 0.550–0.884)-fold risk of disease progression (Table 3). LANR presented significant associations with PFS among patients in age (≥65 yr), gender (female), tumor locations (rectum), differentiation (medium), and TNM stages (III/IV) (Supplementary Figure 5). Combining the area under the curve and the results of multivariate cox regression, we found that LANR is also a valuable new prognostic indicator in 5-year progression-free survival.

**Table 3:** Univariate and multivariate cox regression analysis of progression-free survival in patients with colorectal cancer.

	**AUC**	**Cut-Point**	**Univariate**		Multivariate^*^	
			**HR (95%CI)**	***P***	**HR (95%CI)**	***P***
ALB (G/L)	0.5462	43.3	0.730 (0.572–0.931)	0.0113	0.800 (0.624–1.026)	0.0789
Neu (10^9^/L)	0.5754	3.47	1.680 (1.326–2.130)	<0.0001	1.450 (1.141–1.843)	0.0024
Lym (10^9^/L)	0.5720	1.16	0.657 (0.517–0.835)	0.0006	0.727 (0.570–0.926)	0.0099
LANR	0.5963	16.81	0.597 (0.472–0.755)	<0.0001	0.697 (0.550–0.884)	0.0029
TNM stage^#^			3.568 (2.721–4.678)	<0.0001	3.243 (2.467–4.263)	<0.0001
Differentiation			0.573 (0.455–0.722)	<0.0001	0.623 (0.493–0.788)	<0.0001
Circumferential margin			3.968 (2.222–7.084)	<0.0001	4.545 (2.533–8.156)	<0.0001
Vascular tumor thrombus			1.527 (1.202–1.940)	0.0005	1.449 (1.139–1.843)	0.0025
Nerve invasion			1.642 (1.273–2.117)	0.0001	1.569 (1.216–2.024)	0.0005
Chemotherapy			2.035 (1.523–2.719)	<0.0001	2.015 (1.506–2.696)	<0.0001
Radiotherapy			2.173 (1.460–3.234)	0.0001	1.925 (1.290–2.871)	0.0013

HR, Hazard Ratio; CI, Confidence Interval; AUC, Area under the ROC Curve; Lym, lymphocyte; ALB, albumin; Neu, neutrophils; LANR, Lym*Alb/Neu.

* *Multivariate cox regression models included age, TNM stage, differentiation, circumferential margin, vascular tumor thrombus, nerve invasion, chemotherapy, radiotherapy, and the clinical indicators for mutual adjustment*.

# *TNM Stage adopted binary classification (I/II vs. III/IV)*.

*Reference group: TNM (I/II); Differentiation (Low); Circumferential margin (No); Vascular tumor thrombus (No); Nerve invasion (No); Chemotherapy (No); Radiotherapy (No)*.

## Discussion

In this study, we developed a novel indicator—LANR, which was based on lymphocytes, neutrophils, and albumin, and the results showed that the indicator was significantly correlated with the prognosis of colorectal cancer patients. And to our knowledge, this is the first study to investigate the prognostic value of LANR in colorectal cancer patients.

The development of cancer and its response to treatment are strongly influenced by innate and adaptive immunity, which promote or reduces tumorigenesis and may have opposite effects on the outcome of treatment. At the same time, chronic inflammation promote tumor development, progression, metastatic spread and treatment resistance (24). In addition, studies have shown that systemic inflammation is a marker of poor prognosis present in around 20–40% of colorectal cancer patients (25). Neutrophils are the main circulating granulocytes in humans. They reflect the state of host inflammation and are a hallmark of cancer (26). Neutrophils are involved in different stages of the carcinogenic process, including tumorigenesis, growth, proliferation, or metastatic spread (27, 28). It promotes tumorigenesis by releasing reactive oxygen species (ROS), reactive nitrogen (RNS), or proteases (29), promotes tumor proliferation by weakening the immune system, and also promotes metastatic spread by inhibiting natural killing function and promoting tumor cell extravasation (30). Studies have shown that neutrophils are associated with poor prognosis (31). And the higher the neutrophil level, the higher the risk of progression and death (20, 32). As one of the main cells of human immunity, lymphocytes can produce an immune response to tumor cells, and the decrease of the lymphocytes leads to a decrease in the body's ability to inhibit tumors (33). At the same time, lymphocytes participate in cytotoxic cell death and inhibition of tumor cell proliferation and migration (34). As research continues to develop, more and more evidence suggests that lymphocytes play an important role in predicting the prognosis in colorectal cancer (35–37). And high levels of lymphocytes were significantly associated with good tumor behavior and better survival (38). In addition to inflammatory factors, nutritional indicators can also predict complications, recurrence and prognosis in patients with colorectal cancer. Using prognostic nutrition indicators to investigate the nutrition and immune status of patients may be a useful clinical approach (39). Serum albumin is a common indicator of nutritional status (40). Studies have shown that serum albumin levels were significantly related to overall survival (41). And low levels of serum albumin are associated with a poor overall prognosis in patients with colorectal cancer (42, 43). In this study, we combined these valuable indicators to construct a new prognostic indicator that has good prognostic performance for both overall survival (HR: 0.551; 95% CI: 0.393–0.772) and progression-free survival (HR: 0.697; 95% CI: 0.550–0.884). But we also have some limitations. First, the sample size included in the study was relatively small. Second, as a single-center study, the conclusion may be biased. Therefore, a lot of research is needed to further confirm our findings. In this study, we found a new index that is easily available and has good prognostic performance, which will provide a reference for clinicians and patients to choose a treatment method.

## Data Availability Statement

The raw data supporting the conclusions of this article will be made available by the authors, without undue reservation.

## Ethics Statement

The study was supported by the Ethics Committee and Institutional Review Board of Hubei Cancer Hospital. And all patients provided informed consent.

## Author Contributions

SW and YL contributed to the conception and design of the study, reviewed and edited the manuscript, guarantors of this work, who have full access to all the data in the study, and take responsibility for the integrity of the data and the accuracy of the data analysis. XL, SY, PL, YM, HX, ZY, JH, SW, and YL contributed to the acquisition of data, reviewed, and commented on various versions of the manuscript. XL and SY analyzed the data and wrote the first draft of the manuscript. All authors agree to be responsible for all aspects of the work and give final approval for the submission.

## Conflict of Interest

The authors declare that the research was conducted in the absence of any commercial or financial relationships that could be construed as a potential conflict of interest.
