# Supplementary figures and images for: The Prognostic Value of New Index (LANR) Composed of Pre-operative Lymphocytes, Albumin, and Neutrophils in Patients With Resectable Colorectal Cancer
Source: Front Oncol. 2021 May 25;11:610264. doi: 10.3389/fonc.2021.610264 (PMC8210780; doi:10.3389/fonc.2021.610264)

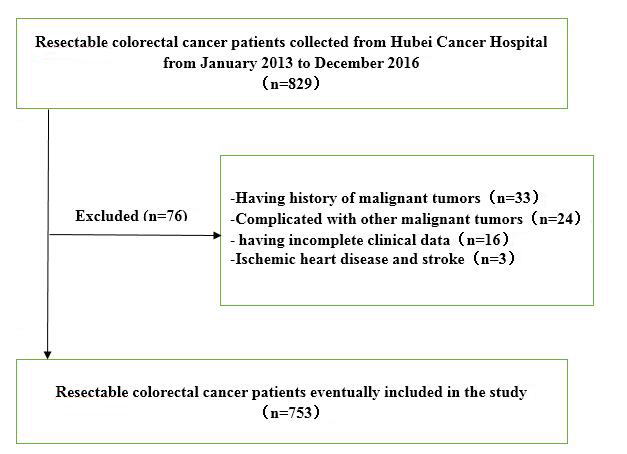

Supplement: Supplementary file 1 [file Image_1.TIF]

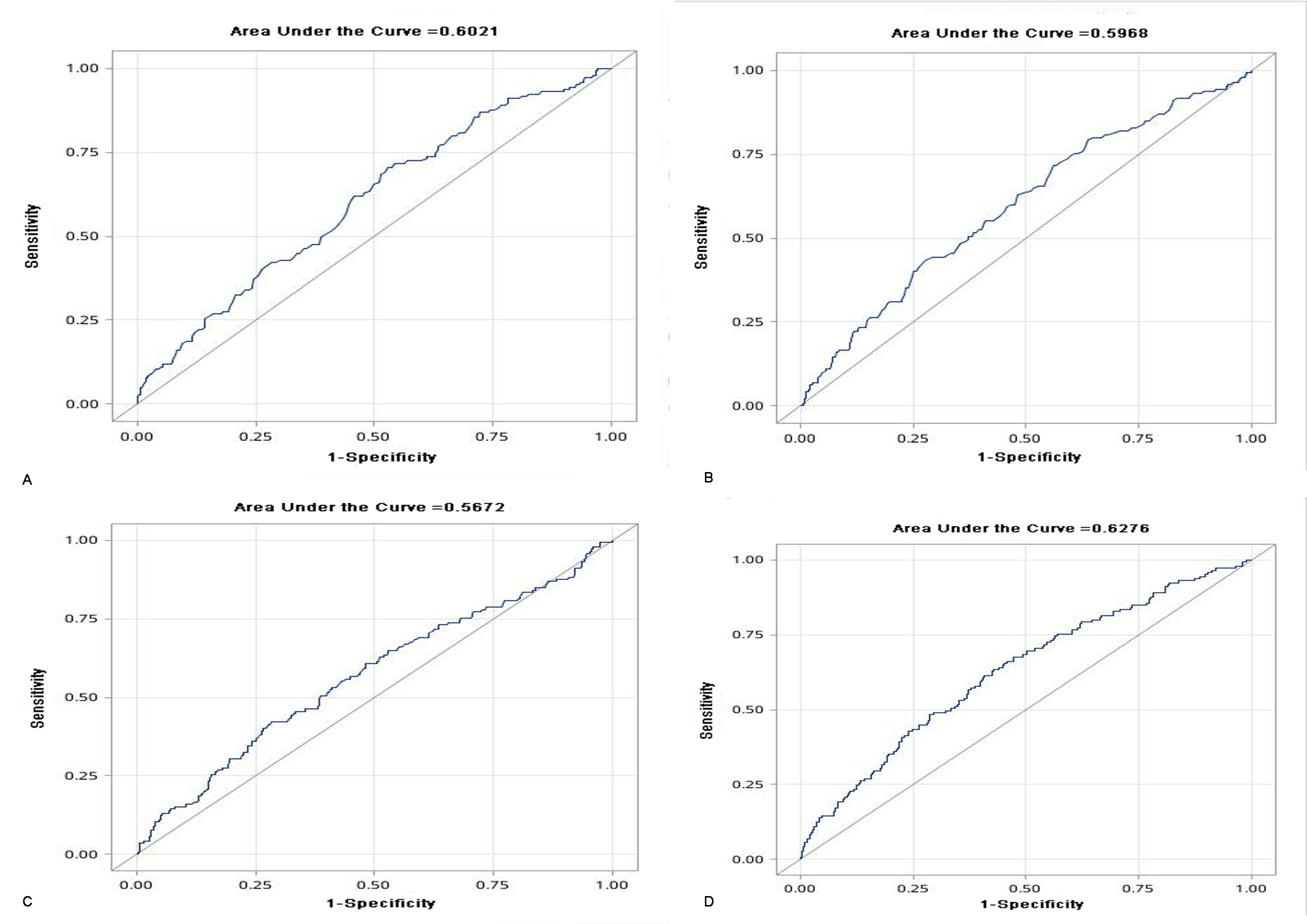

Supplement: Supplementary file 2 [file Image_2.TIF]

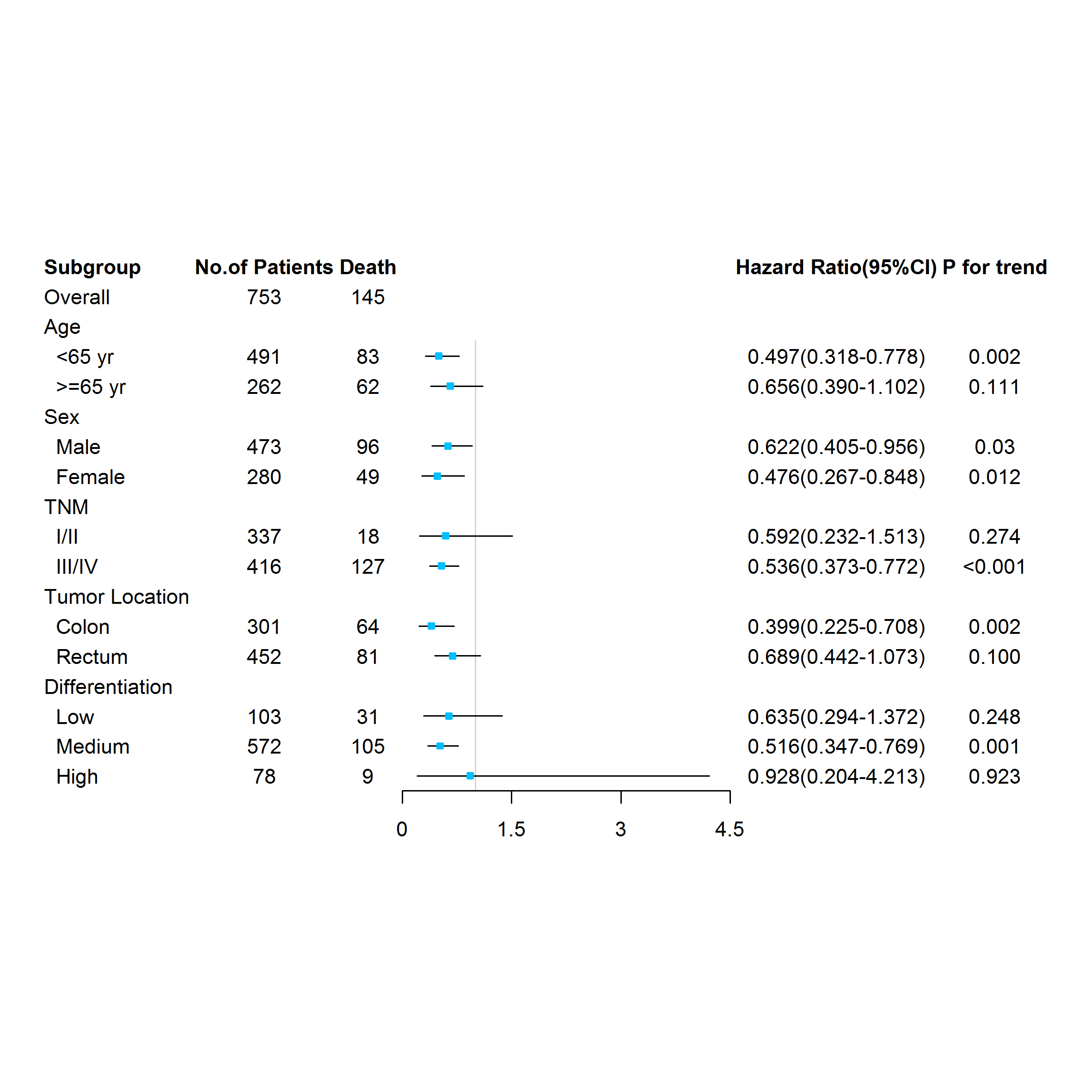

Supplement: Supplementary file 3 [file Image_3.TIF]

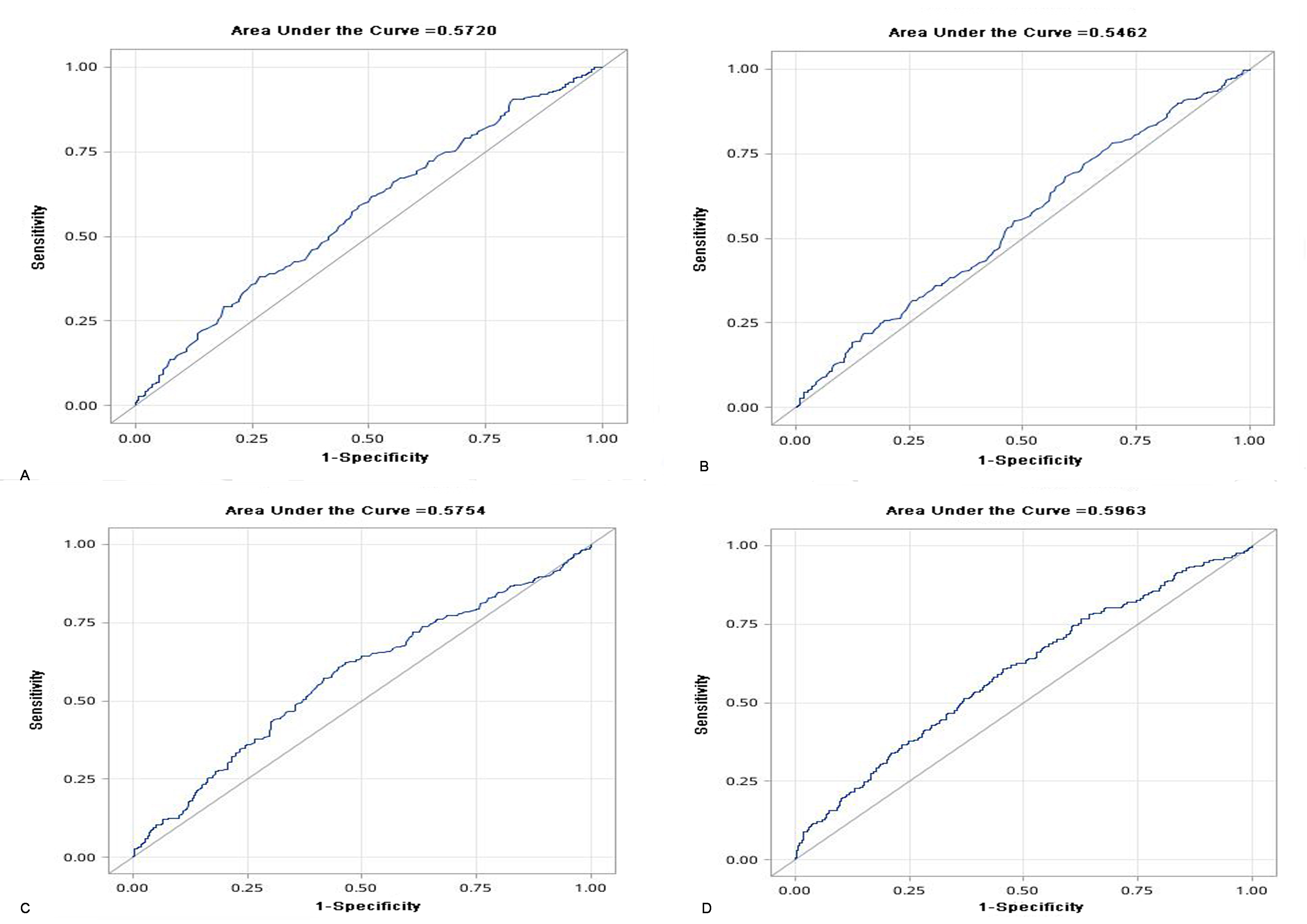

Supplement: Supplementary file 4 [file Image_4.TIF]

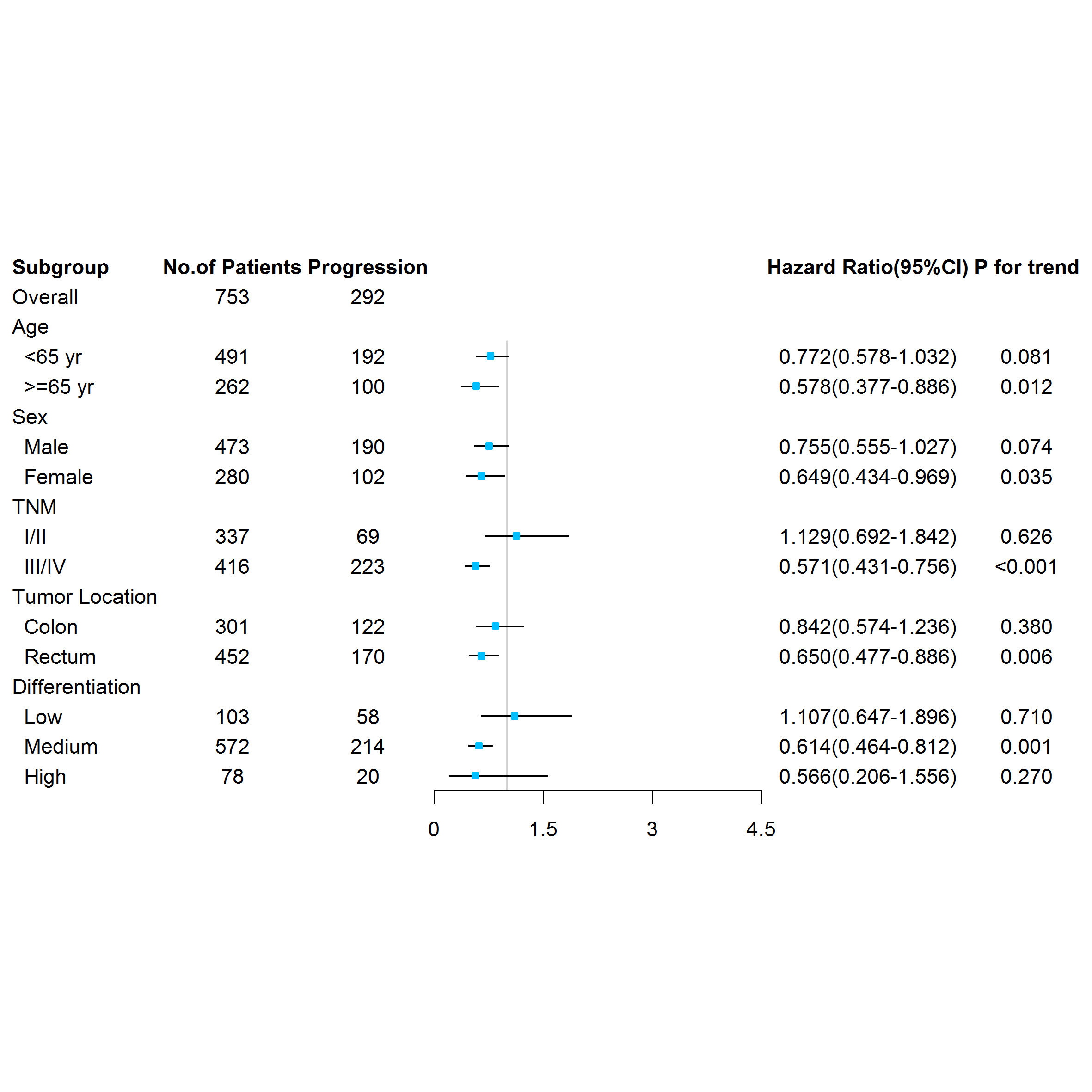

Supplement: Supplementary file 5 [file Image_5.TIF]
