# Supplementary material for: The Prognostic Value of New Index (LANR) Composed of Pre-operative Lymphocytes, Albumin, and Neutrophils in Patients With Resectable Colorectal Cancer
Source: Front Oncol. 2021 May 25;11:610264. doi: 10.3389/fonc.2021.610264 (PMC8210780; doi:10.3389/fonc.2021.610264)
Supplement: Supplementary file 6 [file Data_Sheet_1.docx]

**Table 1_Supp. Relationship between clinicopathological characteristics of overall survival in patients with colorectal cancer and LANR.**

|  |  | LANR value | | *P^*^* |
| --- | --- | --- | --- | --- |
|  |  | Low | High |  |
|  |  | (n=335) (%) | (n=418) (%) |  |
| Age (yr) (mean±SD)^a^ |  | 61(52-68) | 59(52-67) | 0.084 |
| Sex | Male | 227(67.76) | 246(58.85) | 0.012 |
|  | Female | 108(32.24) | 172(41.15) |  |
| Tumor Location | Colon | 158(47.16) | 143(34.21) | <0.001 |
|  | Rectum | 177(52.84) | 275(65.79) |  |
| TNM stage | I | 27(8.06) | 57(13.64) | <0.001 |
|  | II | 96(28.66) | 157(37.56) |  |
|  | III | 121(36.12) | 153(36.60) |  |
|  | IV | 91(27.16) | 51(12.20) |  |
| Tumor size (cm) | d<2 | 4(1.19) | 10(2.39) | 0.476 |
|  | 2≤d<5 | 232(69.25) | 284(67.94) |  |
|  | d≥5 | 99(29.55) | 124(29.67) |  |
| Differentiation | Low | 57(17.01) | 46(11.00) | 0.025 |
|  | Medium | 250(74.63) | 322(77.03) |  |
|  | High | 28(8.36) | 50(11.96) |  |
| Circumferential margin | No | 329(98.21) | 410(98.09) | 0.999 |
|  | Yes | 6(1.79) | 8(1.91) |  |
| Vascular tumor thrombus | No | 217(64.78) | 312(74.64) | 0.004 |
|  | Yes | 118(35.22) | 106(25.36) |  |
| Nerve invasion | No | 254(75.82) | 329(78.71) | 0.381 |
|  | Yes | 81(24.18) | 89(21.29) |  |
| Chemotherapy | No | 92(27.46) | 138(33.01) | 0.111 |
|  | Yes | 243(72.54) | 280(66.99) |  |
| Radiotherapy | No | 312(93.13) | 397(94.98) | 0.348 |
|  | Yes | 23(6.87) | 21(5.02) |  |

LANR, Lym*Alb/Neu.

^*^*P* values were calculated by the Student’s t-test or Wilcoxon test for continuous variables, and the Chi-square test for categorical variables, respectively.

^a^ Age is a continuous variable, the others (Sex, Tumor Location, TNM stage, Tumor size, Differentiation, Circumferential margin, Vascular tumor thrombus, Nerve invasion, Chemotherapy and Radiotherapy) are categorical variables.

**Table 2_Supp. Relationship between clinicopathological characteristics of progression-free survival in patients with colorectal cancer and LANR.**

|  |  | LANR value | | *P^*^* |
| --- | --- | --- | --- | --- |
|  |  | Low | High |  |
|  |  | (n=386) (%) | (n=367) (%) |  |
| Age (yr) (mean±SD)^a^ |  | 62(52-68) | 59(51-66) | 0.008 |
| Sex | Male | 262(67.88) | 211(57.49) | 0.003 |
|  | Female | 124(32.12) | 156(42.51) |  |
| Tumor Location | Colon | 180(46.63) | 121(32.97) | <0.001 |
|  | Rectum | 206(53.37) | 246(67.03) |  |
| TNM stage | I | 30(7.77) | 54(14.71) | <0.001 |
|  | II | 110(28.50) | 143(38.96) |  |
|  | III | 144(37.31) | 130(35.42) |  |
|  | IV | 102(26.42) | 40(10.90) |  |
| Tumor size (cm) | d<2 | 5(1.30) | 9(2.45) | 0.495 |
|  | 2≤d<5 | 265(68.65) | 251(68.39) |  |
|  | d≥5 | 116(30.05) | 107 (29.16) |  |
| Differentiation | Low | 63(16.32) | 40(10.90) | 0.037 |
|  | Medium | 290(75.13) | 282(76.84) |  |
|  | High | 33(8.55) | 45(12.26) |  |
| Circumferential margin | No | 380(98.45) | 359(97.82) | 0.596 |
|  | Yes | 6(1.55) | 8(2.18) |  |
| Vascular tumor thrombus | No | 257(66.58) | 272(74.11) | 0.026 |
|  | Yes | 129(33.42) | 95(25.89) |  |
| Nerve invasion | No | 292(75.65) | 291(79.29) | 0.257 |
|  | Yes | 94(24.35) | 76(20.71) |  |
| Chemotherapy | No | 107(27.72) | 123(33.51) | 0.096 |
|  | Yes | 279(72.28) | 244(66.49) |  |
| Radiotherapy | No | 359(93.01) | 350(95.37) | 0.213 |
|  | Yes | 27(6.99) | 17(4.63) |  |

LANR, Lym*Alb/Neu.

^*^*P* values were calculated by the Student’s t-test or Wilcoxon test for continuous variables, and the Chi-square test for categorical variables, respectively.

^a^ Age is a continuous variable, the others (Sex, Tumor Location, TNM stage, Tumor size, Differentiation, Circumferential margin, Vascular tumor thrombus, Nerve invasion, Chemotherapy and Radiotherapy) are categorical variables.

**Supplementary figure legends**

**Figure 1_Supp.** The flow chart on the inclusion and exclusion criteria of participants.

**Figure 2_Supp.** The ROC curve for overall survival of Lym, Alb, Neu and LANR. A: The ROC curve for overall survival of Lym. B: The ROC curve for overall survival of Alb. C: The ROC curve for overall survival of Neu. D: The ROC curve for overall survival of LANR.

**Figure 3_Supp.** Forest plots of the associations of LANR with the overall survival of colorectal cancer patients in different subgroups.

**Figure 4_Supp.** The ROC curve for progression-free survival of Lym, Alb, Neu and LANR. A: The ROC curve for progression-free survival of Lym. B: The ROC curve for progression-free survival of Alb. C: The ROC curve for progression-free survival of Neu. D: The ROC curve for progression-free survival of LANR.

**Figure 5_Supp.** Forest plots of the associations of LANR with the progression-free survival of colorectal cancer patients in different subgroups.
